# MIRLET7BHG promotes hepatocellular carcinoma progression by activating hepatic stellate cells through exosomal SMO to trigger Hedgehog pathway

**DOI:** 10.1038/s41419-021-03494-1

**Published:** 2021-03-26

**Authors:** Yunhong Xia, Lu Zhen, Hongxia Li, Shuomin Wang, Sun Chen, Chongchong Wang, Xiaoyu Yang

**Affiliations:** 1 Department of Oncology, the Fourth Affiliated Hospital, Anhui Medical University, Hefei, Anhui 230022 China; 2 Department of General Surgery, the Fourth Affiliated Hospital, Anhui Medical University, Hefei, Anhui 230022 China; 3 Department of Oncology, Hefei First People’s Hospital, Anhui Medical University, Hefei, 230022 China

**Keywords:** Cancer stem cells, Cell biology

## Abstract

Hepatocellular carcinoma (HCC), commonly caused by liver fibrosis, is a global challenge with high morbidity. Activation of hepatic stellate cells (HSCs) contributes to hepatic fibrosis. Exosomes are small vesicles that play a significant role in cell-to-cell communication. Smoothened (SMO) is the key signal transducer for Hedgehog pathway. This study was designed to study the function and underlying mechanism of SMO in HSC activation. Functional assays including 5-Ethynyl-2´-deoxyuridine, colony formation, wound healing, transwell, and sphere formation assays disclosed the function of SMO. Western blot analysis of exosome biomarkers, immunofluorescence staining assay, electron microscope, and flow cytometry revealed the existence of exosomes. Bioinformatics analyses and mechanistic assays uncovered the interplays between RNAs. Nude mice xenograft model was established to evaluate HCC tumor growth. We uncovered that SMO was an oncogene in HCC cells and was low-expressed in quiescent HSCs. Then, SMO was upregulated in HSCs cultured with HCC cells-conditioned medium. Next, it was revealed that HCC cells-derived exosomes activated HSCs by transmitting SMO to HSCs. Subsequently, we recognized that microRNA let-7b host gene (MIRLET7BHG) served as the competing endogenous RNA against miR-330-5p to upregulate SMO. In turn, SMO induced hedgehog pathway to promote GLI family zinc finger 1 (Gli1), leading to transcriptional activation of MIRLET7BHG in activated HSCs. In summary, this study demonstrated that Gli1-induced MIRLET7BHG facilitated HCC by activating HSCs through exosomal SMO to stimulate hedgehog pathway, providing a new road for HCC treatment.

## Introduction

Hepatocellular carcinoma (HCC) is the third most common cancer worldwide.^1^ A great number of HCC cases are caused by liver fibrosis or cirrhosis.^2^ Hepatic stellate cells (HSCs) play a major role in hepatic fibrosis and have received much attention over years. Normally, HSCs are at quiescent stage and have many cytoplasmic lipid droplets to store vitamin A.^3^ However, if injuries occur, HSCs are changed from the quiescent stage to the active stage, and the activated HSCs finally differentiate to myofibroblast-like cells. Of note, activated HSCs express α‐SMA and release excessive extracellular matrix (ECM) including collagens and cellular fibronectin to advance hepatic fibrosis.^4,5^

Since HSCs are important participants in the pathogenesis of hepatic fibrosis, a growing number of studies have focused on the molecular mechanisms underlying HSC activation. Park YJ et al. confirmed that (-)-Catechin-7-O-β-d-Apiofuranoside (C7A) inhibits the activation of HSCs by inactivating STAT3 pathway.^6^ Zhang K et al.^7^ uncovered that SCARNA10 contributes to liver fibrosis by inducing HSCs activation. Hsu YL et al. pointed out that bone-marrow-derived cells-derived extracellular vesicles (EVs) induces miR-92a upregulation to promote the activation of HSCs.^8^ Liu W et al.^9^ suggested that circ-PWWP2A induces HSC activation and proliferation via sponging miR-203 and miR-223 to respectively upregulate Fstl1 and TLR4.

Exosomes, a group of small EVs with the size of 50–100 nM, are important media for cell-to-cell communication. Under physiological and pathological conditions, exosomes can be released from cells to the extracellular environment upon the fusion of multivesicular bodies and plasma membrane. Tumor cells-derived exosomes share genetic information or functional proteins, which enable them to serve as crucial mediators between tumor cells and other cells in the tumor microenvironment.^10^ Liang ZX^11^ revealed that colorectal cancer cells-derived exosomes transmit RPPH1 to facilitate macrophage M2 polarization, thus promoting cancer cell proliferation and migration. Huang L et al.^12^ revealed that PCAT1 exists in esophageal squamous cell carcinoma cells-derived exosomes and promotes cell growth through exosomes. HCC cells-derived exosomes activate nuclear factor kappa b signaling and induce pro-inflammatory factors to remodel macrophages, resulting in M2 polarization of tumor-associated macrophages.^13^ Besides, exosomes have been reported to play a crucial role in the activation of HSC via multiple mechanisms. For instance, HCC cells-derived exosomal miRNA-21 converts HSCs to cancer-associated fibroblasts and contributes to tumor development.^14^

Long non-coding RNAs (lncRNAs) are longer than 200 nucleotides and are incapable of encoding proteins. Dysregulation of lncRNAs contributes to the occurrence and development of HCC.^15–17^ It has also been revealed that lncRNAs exert significant functions in the activation of HSCs. Linc-SCRG1 exhibits a promoting effect on HSC activation and contributes to human liver fibrosis.^18^ LincRNA-p21 sponges miR-17-5p and miR-17-5p to inhibit Wnt/β-Catenin pathway, thus inactivating HSCs.^19^ MEG3 blocks the activation of HSCs and alleviates liver fibrosis by interacting with miR-212 and smoothened (SMO) protein.^20^ Moreover, lncRNAs have been widely reported to participate in the competitive endogenous RNA (ceRNA) network. LncRNAs bind with microRNAs (miRNAs) to antagonize the suppression of miRNAs on target genes. The ceRNA pattern has also been widely reported in the development of HCC or liver fibrosis.^21–24^

SMO is a key signal transducer in hedgehog pathway, which is closely associated with cell proliferation, apoptosis, migration, and invasion in various tumors,^25,26^ including HCC.^27^ Present study started from SMO and probed into its function and upstream mechanism in activating HSCs.

## Materials and methods

### Cell lines and treatment

HCC cell lines (Hep 3B, LM3, 97H, and Huh-7) and HSC cell line (LX2) used for this study were procured from Shanghai Pituo Biological Technology Company Ltd (Shanghai, China) and maintained in a humidified incubator supplied with 5% CO_2_ at 37°C. Moreover, 10% fetal bovine serum and 1% penicillin/streptavidin were acquired from Gibco (Rockville, MD, USA) and served as the medium supplements of Dulbecco’s Modified Eagle Medium (DMEM) and Roswell Park Memorial Institute (RPMI-1640) (Gibco). LM3, 97H, Huh-7, and LX2 cells were cultivated in RPMI-1640, whereas Hep 3B cells were grown in DMEM. To activate Hedgehog pathway, cells were treated with 5 µM of SMO agonist (SAG; Millipore, Bedford, MA, USA).

### Quantitative real-time polymerase chain reaction (qRT-PCR)

The extracted total RNA was prepared by treating the cultured cells with TRIzol Reagent (Invitrogen, Carlsbad, CA, USA) as per the instruction. 500 ng of total RNA was converted into cDNA in the presence of PrimeScript Reverse Transcriptase Kit (Takara, Kyoto, Japan). qRT-PCR was conducted for determining gene expression with SYBR Green PCR Kit (Takara). All data were normalized to U6 or GAPDH based on 2^−ΔΔCT^ method.

### Cell transfection

The shRNAs specifically targeting SMO, miRNA let-7b host gene (MIRLET7BHG), GLI family zinc finger 1 (Gli1), and their corresponding control sh-NC, were synthesized by Genechem (Shanghai, China). For overexpressing SMO or Gli1, their full-length cDNA sequences were separately inserted into pcDNA3.1 vectors (Invitrogen). The empty vectors were used in control group. The miR-330-5p mimics and NC mimics were designed by GenePharma (Shanghai, China). The transfections of the above plasmids into the indicated cells (LX2, LM3, 97H) were accomplished with Lipofectamine 3000 (Invitrogen), in line with the instruction. Two days later, the transfected cells were collected for next use.

### EdU assay

5-Ethynyl-2´-deoxyuridine (EdU) assay was completed by use of BeyoClick™ EdU Cell Proliferation Kit (Beyotime, Shanghai, China) and Alexa Fluor 594. LX2, LM3, or 97H cells were subjected to PBS washing and then incubated with EdU medium for 2 h at 37°C. After that, cells were fixed for 30 min using 4% paraformaldehyde (PFA) prior to staining in 4’,6-diamidino-2-phenylindole (DAPI) solution, and finally observed under the inverted microscope (Olympus, Tokyo, Japan). EdU assay was undertaken three times.

### Colony formation assay

LX2, LM3, or 97H cells at logarithmic phases were seeded at 500/well into six-well plates and cultivated for 14 days. After that, the resulting colonies were treated with 4% PFA for 30 min of fixation followed by 5 min of staining in crystal violet (0.5%). Colonies were counted manually. Three bio-repeats were required for this assay.

### Wound-healing assay

The cultured cells were plated in six-well plates until reaching 90% confluence. Then, the artificial scratch was made in each well by use of 200-μL pipette tip. The wound closure images were captured at 0 and 24 h post incubation in a serum-free medium for assessing the cell migration ability. Three independently conducted assays were conducted for analysis.

### Transwell assay

For invasion assay, the 24-well transwell inserts were coated with 30 μg of Matrigel (BD Biosciences, Franklin Lakes, NJ, USA) before use. Migration assay was undertaken similarly without Matrigel coating. The suspension of transfected cells (1 × 10^5^) were prepared and placed into the upper chamber in a serum-free medium, whereas the lower chamber was supplemented with 500 μl of complete culture medium (CM). Following 24 h of culturing, cells in the lower chambers were fixed using 4% PFA prior to crystal violet staining. Then, the stained cells were randomly counted in five fields under a light microscope (Olympus). Transwell assays were performed in triplicate.

### Western blot

RIPA lysis buffer was first employed to lyse the indicated cells, and then the collected total protein was separated by sodium dodecyl sulfate polyacrylamide gel electrophoresis (12%) and shifted to polyvinylidene difluoride membranes. After being blocked by 5% nonfat milk, the membranes were probed all night at 4°C with primary antibodies, including the loading control GAPDH (ab8245, 1/1000; Abcam, Cambridge, MA, USA), Nanog (ab109250, 1/1000; Abcam), OCT4 (ab181557, 1/1000; Abcam), N-cadherin (ab76057, 1/1000; Abcam), E-cadherin (ab76055, 1/1000; Abcam), CD9 (ab92726, 1/2000; Abcam), HSP70 (ab2787, 1/1000; Abcam), CD81 (ab109201, 1/1000; Abcam), TSG101 (ab125011, 1/1000; Abcam), α-smooth muscle actin (α-SMA) (#19245 S, 1/1000; Cell Signaling Technology, Danvers, MA, USA), fibroblast activation protein (FAP) (#66562 S, 1/1000; Cell Signaling Technology), SMO (ab8969, 1/1000; Abcam), Gli1 (ab134906, 1/1000; Abcam) and collagen type I (ab34710, 1/1000; Abcam). After processing with tris-buffered saline and polysorbate 20 thrice, the membranes were subjected to cultivation with corresponding horseradish peroxidase-labeled secondary antibodies at room temperature for 2 h. The protein signals were examined using the ECL luminous liquid (Pierce, Rockford, IL, USA). Western blot was conducted in triplicate three times.

### Sphere formation assay

The indicated cells were placed to 96-well ultralow attachment plates (Corning Inc., New York, NY, USA) at 10 cells/well. Following 7 days of cell culture in serum-free medium, the spheres, defined as cell clusters with >50 mm, were counted and imaged under a light microscope. Bio-triple assays were required.

### Isolation and analysis of exosomes

For exosome isolation, the equal numbers of indicated cells were planted to 10-cm plates and maintained in RPMI-1640 with serum all night and then centrifuged at 12,000 × *g*. The CM was collected after 48 h and filtrated by 0.22-μm filters (Millipore). The exosomes in indicated CMs were isolated by ultracentrifugation, as previously described.^28^ The isolated exosomes were observed using Philips CM120 BioTwin transmission electron microscope (FEI Company, Hillsboro, OR, USA), and their size was analyzed by flow cytometry (BD Biosciences, San Jose, CA).

### Exosomes tracking by immunofluorescence (IF) staining

The isolated exosomes were treated with PKH67-labeling kit (Beyotime, Shanghai, China) for exosome-tracking assay. The recipient cells were treated with PKH67-labeled exosomes and then stained with DAPI after fixing using 4% PFA. Finally, cells were observed under a fluorescence microscope. Bio-triple assays were required.

### ELISA assay

The enzyme-linked immunosorbent assay (ELISA) assay was implemented as per the described protocol.^29^ In brief, the amount of pro-collagen I α1 in the conditioned medium (CM) of quiescent or exosomes-activated LX2 cells was monitored by use of a Duoset® ELISA kit (R&D Systems). CM was previously stored at −20°C before use. The enzyme activity was tested with an ELISA microplate reader (POLARstar Omega, BMG LABTECH, Champigny sur Marne, France). This assay was conducted in triplicate three times.

### RNA pull-down assay

Based on the established protocol (Thermo Fisher Scientific, Waltham, MA, USA), RNA pull-down assay was undertaken with Pierce Magnetic RNA-Protein Pull-Down Kit. The cell lysates were prepared to mix overnight with the biotinylated probes for SMO or miR-330-5p and then with magnetic beads for 1 h. The final RNA-RNA mixture was analyzed using qRT-PCR. The experiment was implemented in triplicate.

### Luciferase reporter assay

Full-length MIRLET7BHG or SMO 3′-UTR fragments covering wild-type and mutated miR-330-5p-binding sites were synthesized and cloned to the pmirGLO dual-luciferase vectors (Promega, Madison, WI, USA), so that MIRLET7BHG-WT/MUT and SMO-WT/MUT were obtained. The acquired constructs were then co-transfected with miR-330-5p mimics (or NC mimics) into LM3-EXO/LX2 and 97H-EXO/LX2 cells. For promoter analysis, cells were co-transfected with pGL3-basic vector (Promega) containing indicated MIRLET7BHG promoter (wild-type, site 1-mutated, site 2-mutated or site 1/2-mutated) and pcDNA3.1-Gli1 (or pcDNA3.1). Forty-eight hours later, The luciferase activity was studied by dual-luciferase reporter assay system (Promega). Assays were conducted thrice.

### Subcellular fraction

LM3-EXO/LX2 and 97H-EXO/LX2 cells (1 × 10^6^) were treated with precooled PBS and then lysed with cell fractionation buffer. After that, samples were centrifuged, and the supernatant was stored as a cytoplasmic fraction. Then, cell disruption buffer was added to obtain nuclear fraction. qRT-PCR was conducted for quantifying the MIRLET7BHG content in both fractions, with U6 as the nuclear index and GAPDH as the cytoplasmic index. Assay was repeated independently three times.

### RNA immunoprecipitation

By employing the Magna RIP™ RNA-Binding Protein Immunoprecipitation Kit (Millipore), RNA immunoprecipitation (RIP) assay was conducted with human Ago2 antibody and control IgG antibody (Millipore). The cell lysates were processed with RIP buffer containing antibody-bound magnetic beads. The immunoprecipitated RNAs were extracted from beads and then purified for qRT-PCR analysis. RIP assay was undertaken in triplicate.

### Chromatin immunoprecipitation

By applying the EZ ChIP™ Chromatin Immunoprecipitation Kit (Millipore), chromatin immunoprecipitation (ChIP) assay was undertaken in LM3-EXO/LX2 and 97H-EXO/LX2 cells. Samples were fixed for 15 min to maintain the cross-link, followed by processing by ultrasonic for shearing DNA to 500-bp of fragments. Immunoprecipitation was conducted for 6 h with control IgG antibody or Gli1 antibody, in the presence of magnetic beads, followed by qRT-PCR analysis of the precipitated fragments. This assay was performed in triplicate.

### In vivo tumor formation assay

The animal-related protocol was approved by the Institutional Animal Care and Use Committee of the Fourth Affiliated Hospital, Anhui Medical University. The 6-week-old male nude mice, weighing 16–20 g, were commercially acquired from Slac Laboratories (Shanghai, China) and then housed under specific pathogen-free-grade. In vivo tumor formation assay was undertaken via subcutaneous injection of 5 × 10^6^ LM3-EXO/LX2 and 97H-EXO/LX2 cells into nude mice. The volume of tumors was monitored every 4 days. Twenty-eight days later, the mice were killed prior to collecting tumors for weight assessment and immunohistochemistry (IHC) analysis.

### Immunohistochemistry

The paraffin-embedded tumor tissues from in vivo assay were cut into 4 μm and the sections were fixed for IHC analysis. The specific antibody to Ki-67 (#9449) or PCNA (#13110 S) was procured from Cell Signaling Technology and utilized as instructed. The assay was run in triplicate.

### Statistical analyses

Each procedure of all experiments was undertaken in triplicate. The data were exhibited as mean ± standard deviation (SD) and analyzed using GraphPad PRISM 6 (GraphPad, San Diego, CA). Student’s *t* test or one-way analysis of variance was employed for analyzing the difference between groups, with a significant level set as *p* < 0.05.

## Results

### SMO facilitated HCC cell proliferation, migration, invasion, EMT process, and stemness characteristic

**Fig. 1:**
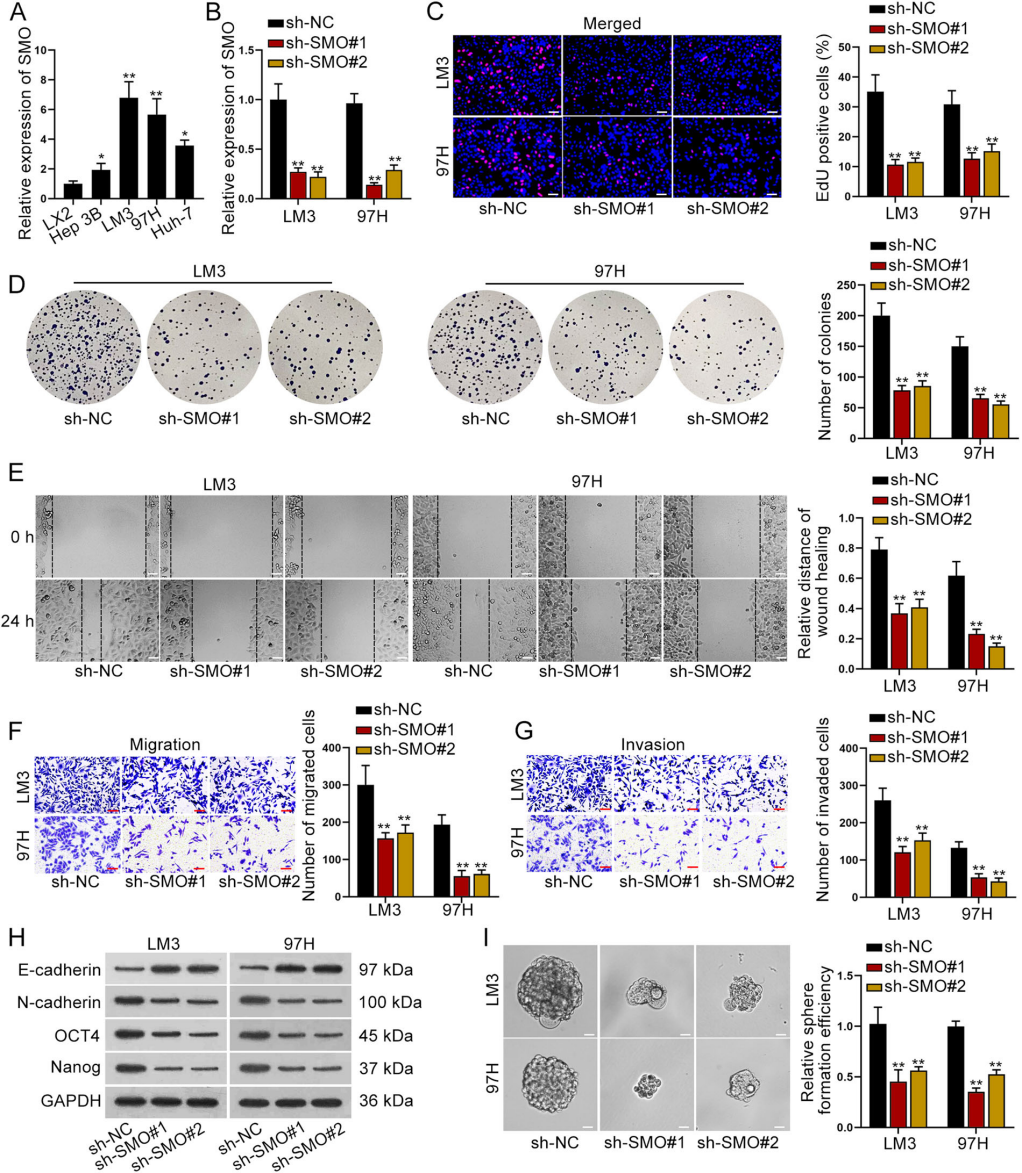
SMO facilitated HCC cell proliferation, migration, invasion, EMT process, and stemness characteristic. **A** qRT-PCR revealed the expression level of SMO in HCC cell lines (Hep 3B, LM3, 97H, and Huh-7) and HSC cell line (LX2). **B** qRT-PCR verified the depletion efficiency of SMO in LM3 and 97H cells. **C**–**D** EdU (bar value = 100 μm) and colony formation assay revealed in the proliferation of HCC cells under the depletion of SMO or not. **E** Wound-healing assay showed the migration ability of HCC cells with or without downregulated SMO (bar value = 80 μm). **F**–**G** Transwell assay revealed HCC cell migration and invasion ability in response to SMO silence (bar value = 50 μm). **H** Western blot detected the expression of E-cadherin, N-cadherin, OCT4, and Nanog in HCC cells transfected with sh-NC or sh-SMO#1/2. **I** Sphere formation assay revealed the sphere formation efficiency of HCC cells after silencing SMO (bar value = 50 μm). *P < 0.05, **P < 0.01.

The initial step of this study was to examine the expression level of SMO in HCC cell lines (Hep 3B, LM3, 97H, and Huh-7) and an HSC cell line (LX2). As revealed by qRT-PCR, SMO level was higher in HCC cells than that in HSC cells (Fig. 1A). As LM3 and 97H cells expressed highest SMO, they were used for the next assays. Then, we knocked down SMO in LM3 and 97H cells by transfection with sh-SMO#1/2 for the following loss-of-function assays (Fig. 1B). EdU staining assay disclosed that the number of EdU-positive cells was significantly reduced by silencing SMO in LM3 and 97H cells (Fig. 1C). Besides, colony formation assay indicated that the number of colonies was also visibly declined when SMO was inhibited in LM3 and 97H cells (Fig. 1D). Then, wound-healing assay showed that the relative distance of wound healing was shortened by knockdown of SMO (Fig. 1E). Furthermore, the transwell assay uncovered that cell migration and invasion capacities were remarkably reduced in LM3 and 97H cells under SMO depletion (Fig. 1F, G). Moreover, the changes in epithelial–mesenchymal transition (EMT) biomarkers (E-cadherin and N-cadherin) and stemness biomarkers (OCT4 and Nanog) were also examined. As detected by western blot, the expression of N-cadherin, OCT4 and Nanog was decreased, whereas that of E-cadherin was increased in both cells due to downregulation of SMO (Fig. 1H). Sphere formation assay also revealed the attenuated sphere formation efficiency in SMO-silenced LM3 and 97H cells (Fig. 1I). Based on these data, we drew a conclusion that SMO contributed to cell proliferation, migration, invasion, EMT process, and stemness characteristic in HCC.

### Tumor-derived exosomes transmitted SMO and activated HSCs

To explore the influence of HCC cells on adjacent HSC cells, we then co-cultured LX2 cells with the CM of LM3 and 97H cells. It was revealed that the proliferation of the co-cultured LX2 cells was accelerated in comparison with control group (Figure S1A–B). Also, LX2 cell migration and invasion were both facilitated after co-culture with the CM of LM3 or 97H cells (Figure S1C–D). Besides, we discovered that the level of E-cadherin was lessened while that of N-cadherin, OCT4, and Nanog was augmented in LX2 cells under co-culture (Figure S1E). Further, the sphere formation ability of LX2 cells was also fortified when treated with CM/LM3 or CM/97H (Figure S1F). All the experimental assays demonstrated that co-cultured LX2 cells exhibited stronger proliferation, migration, invasion, EMT, and stemness characteristics than control LX2 cells. In another word, LX2 was changed from quiescent state to an activated state after co-culture with the CM from HCC cells.

**Fig. 2:**
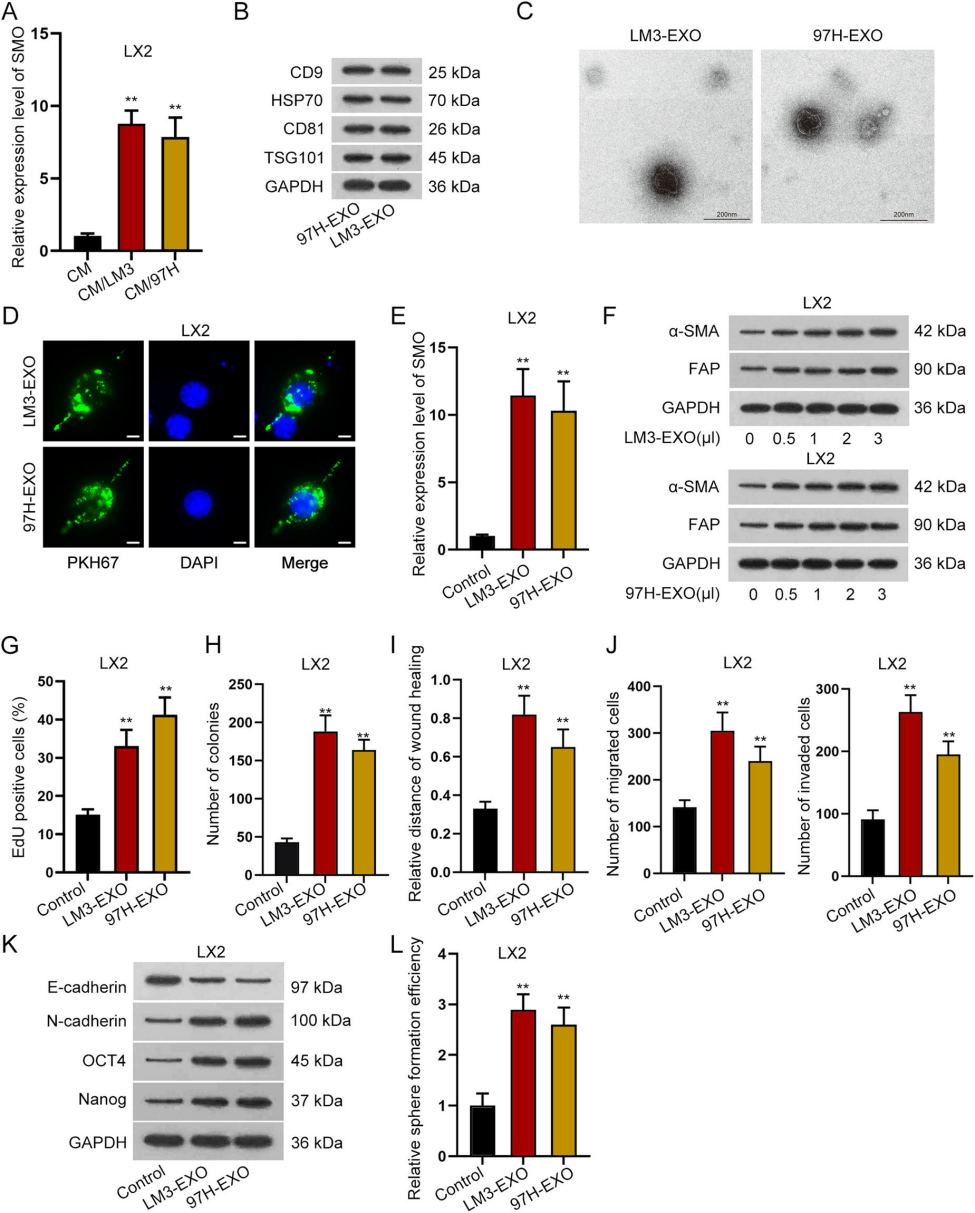
HCC cells-derived exosomes transmitted SMO and activated HSC. **A** qRT-PCR detected the expression of SMO in quiescent HSCs and activated HSCs induced by conditioned medium (CM) of LM3 or 97H cells. **B** Western blot detected the protein level of CD9, HSP70, CD81, and TSG101 in LM3- or 97H-derived exosomes. **C** Electron microscope revealed the images of exosomes. **D** IF staining assay revealed PKH67-labeled exosomes in LX2 cells treated with exosomes from LM3/97H cells (bar value = 15 μm). **E** qRT-PCR detected the expression of SMO in LX2 cells treated with LM3- or 97H-derived exosomes. **F** Western blot assay revealed the protein level of α-SMA and FAP in LX2 cells under the treatment of exosomes at different concentrations. **G**–**J** EdU, colony formation, wound healing, and transwell assays tested cell proliferation, migration, and invasion in LX2 cells with or without the treatment of LM3 or 97H-derived exosomes. **K** Western blot analyzed the levels of E-cadherin, N-cadherin, OCT4, and Nanog in quiescent LX2 cells and exosomes-activated LX2 cells. **L** Sphere formation assay disclosed the effects of LM3- or 97H-derived exosomes on the stemness of LX2 cells. **P < 0.01.

To determine whether such changes were induced by elevated SMO in HCC cells, we then examined SMO level in LX2 cells treated with indicated CMs. As expected, SMO was upregulated in activated LX2 cells than in quiescent LX2 cells (Fig. 2A). As exosomes were reported to activate HSC cells. We wondered if LM3 and 97H cells derived exosomes to promote the activation of HSC cells. In this case, exosomes markers (CD9, HSP70, CD81, and TSG101) were detected and western blot confirmed the high expression of them in the CMs of LM3 and 97H cells (Fig. 2B), proving the existence of exosomes in the CMs. Also, electron microscopy verified that the isolated particles were exosomes (Fig. 2C), and flow cytometry analyzed the size of these exosomes (Figure S2A). Then, PKH67 (green) was used to label exosomes, and IF staining results demonstrated that exosomes were abundantly absorbed by LX2 cells (Fig. 2D). Further, we examined SMO level in LX2 cells treated with exosomes and found that SMO was significantly upregulated in LX2 cells after treating with exosomes from LM3 or 97H cells (Fig. 2E). Intriguingly, SMO expression was increased with the titrated doses of exosomes (Figure S2B). Subsequently, specific markers (α-SMA and FAP) of activated HSCs were detected under different concentration of exosomes. Results manifested that with the increase in the concentration of exosomes, both the expression levels of α-SMA and FAP were gradually boosted in LX2 cells (Fig. 2F). Furthermore, we also detected that the levels of COL1A1 and COL4A1 were increased, along with the strengthened secretion of pro-collagen type I α1 and enhanced level of collagen I, in LX2 cells after treating with exosomes from HCC cells (Figure S2C–E). All these data indicated that LX2 cells were activated under the treatment of exosomes.

We further detected the influence of LM3 and 97H-derived exosomes on the activation of LX2 cells by functional assays. As observed from the outcomes of EdU and colony formation assays, cell proliferation capability was promoted in LX2 cells treated with exosomes (Fig. 2G, H). Data from wound-healing and transwell assays verified that cell migration and invasion were expedited in LX2 cells with the treatment of exosomes (Fig. 2I, J). In addition, western blot analyzed that the expression of N-cadherin, OCT4, Nanog was increased, whereas that of E-cadherin was decreased in LX2 cells treated with exosomes from LM3 or 97H cells (Fig. 2K). Moreover, sphere formation assay revealed the increased sphere formation efficiency after LX2 cells was treated with exosomes (Fig. 2L). Taken together, it was revealed that, under the treatment of exosomes from HCC cells, the activated LX2 cells showed more malignant than quiescent LX2 cells. Based on these data, we concluded that exosomes derived from cancer cells activated HSC cells in HCC.

### SMO facilitated the proliferation, migration, invasion, and EMT of activated HSCs

**Fig. 3:**
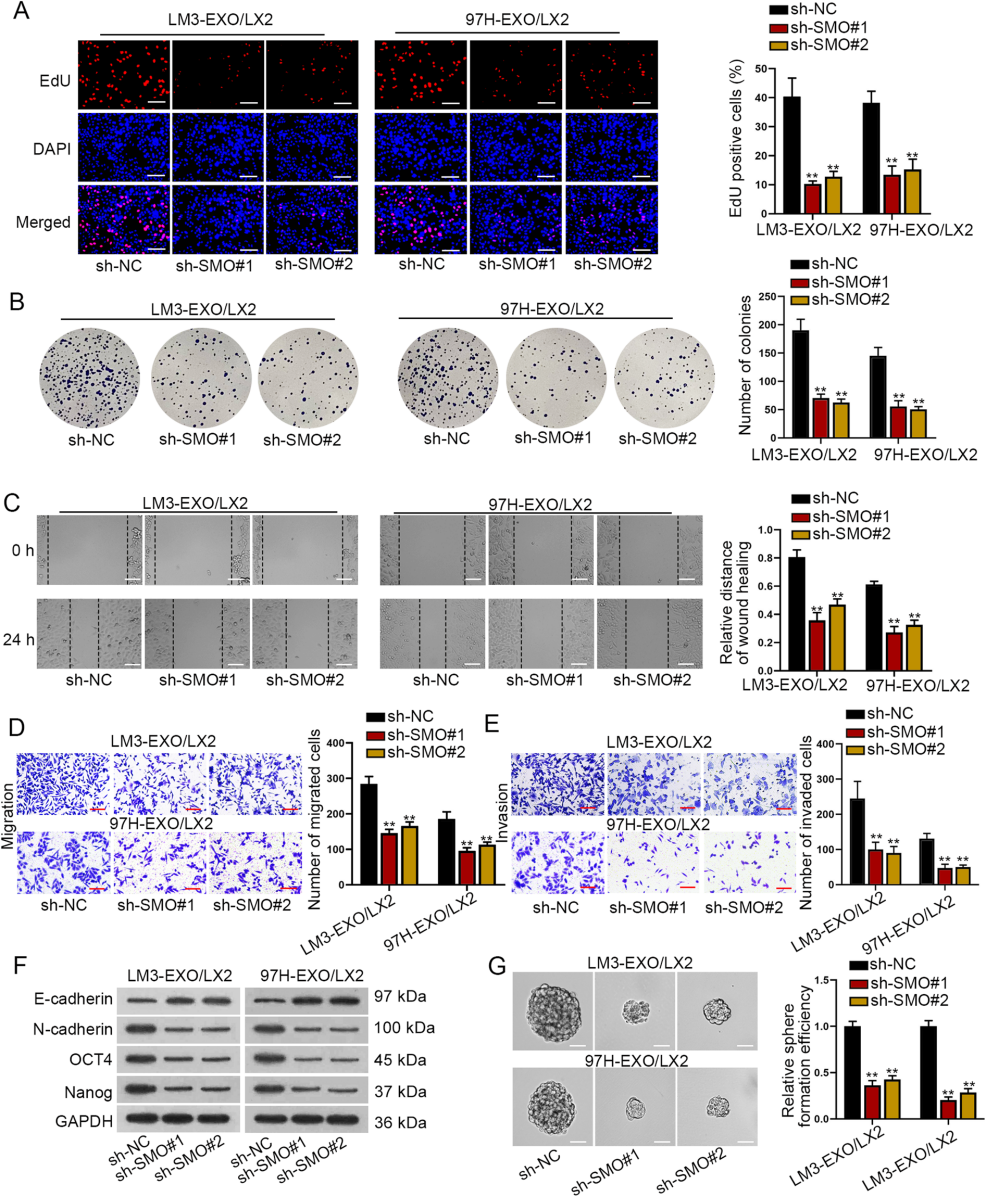
SMO facilitated the proliferation, migration, invasion, EMT, and stemness of activated HSCs. **A**–**B** EdU (bar value = 100 μm) and colony formation assays depicted the proliferation of exosomes-activated HSCs with or without knockdown of SMO. **C**–**E**. Wound-healing (bar value = 80 μm) and transwell assays (bar value = 50 μm) unveiled the influence of silenced SMO on the migration and invasion capacity of exosomes-activated HSCs. **F** Western blot detected the levels of E-cadherin, N-cadherin, OCT4, and Nanog in exosomes-activated HSCs cells with or without SMO deficiency. **G** Sphere formation assay (bar value = 50 μm) uncovered the influence of silenced SMO on the stemness of activated HSCs. **P < 0.01.

Then, we sought to examine the influence of SMO on exosomes-treated HSCs. It was indicated that depletion of SMO significantly impaired the proliferation of LX2 cells even with exosome treatment (Fig. 3A, B). In addition, data from wound-healing and transwell assays disclosed the attenuated migration and invasion of exosomes-cultured LX2 cells under SMO silence (Fig. 3C–E). Meanwhile, western blot analysis results and sphere formation assay data revealed that the EMT process and stemness characteristic were inhibited in such cells by downregulation of SMO (Fig. 3F, G). Based on these data, we deduced that SMO transmitted from HCC cells to HSCs via exosomes promoted the activation of HSCs.

### MIRLET7BHG sponged miR-330-5p to upregulate SMO

**Fig. 4:**
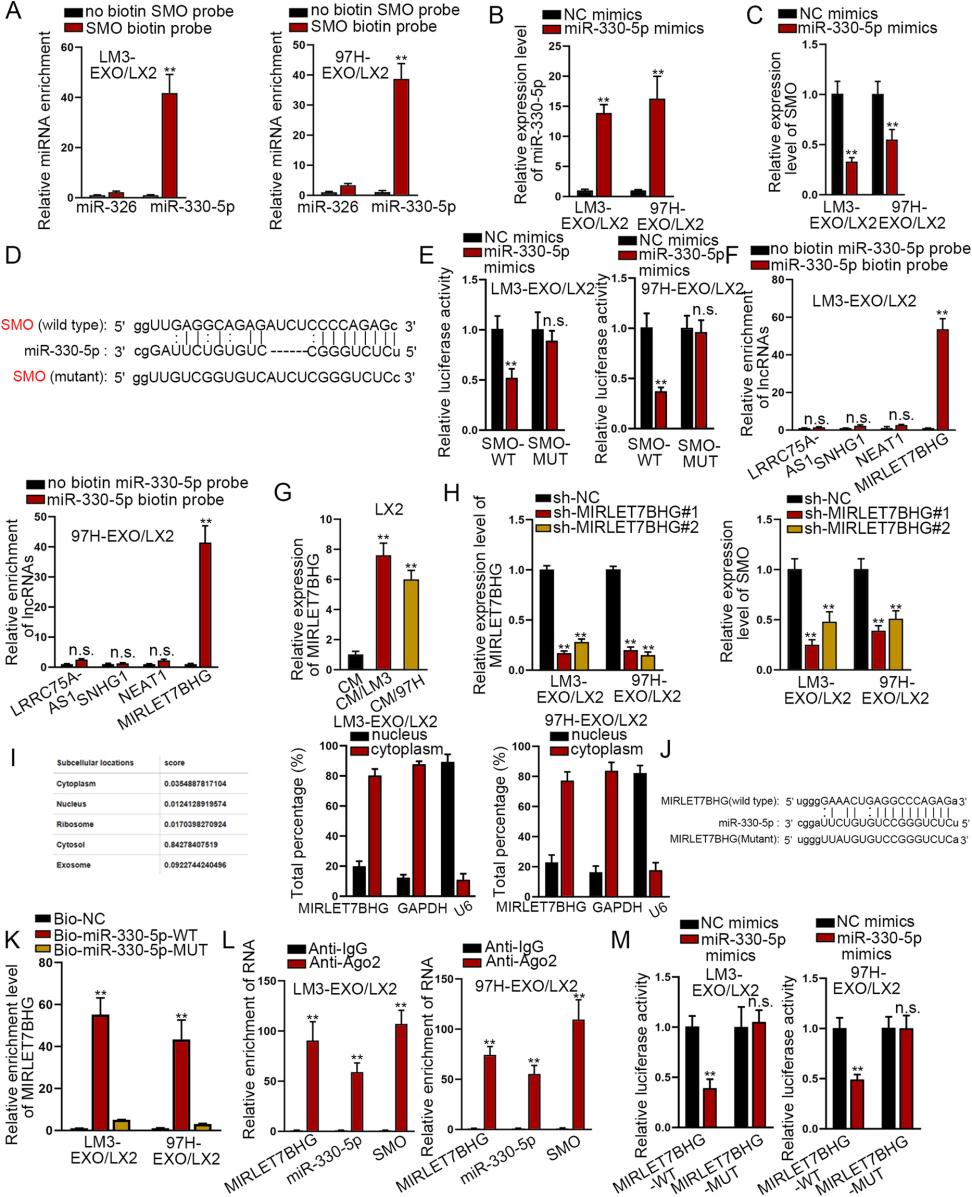
MIRLET7BHG sponged miR-330-5p to upregulate SMO. **A** RNA pull-down assay examined the enrichment of miR-326 and miR-330-5p by biotin-labeled SMO. **B** qRT-PCR examined the overexpression efficiency of miR-330-5p in activated HSCs. **C** qRT-PCR revealed the influence of upregulated miR-330-5p on SMO expression in activated HSCs. **D** Binding sites between SMO and miR-330-5p were predicted by starBase database. **E** Luciferase reporter assay examined the changes in luciferase activity of wild-type and mutant SMO in activated HSCs after overexpressing miR-330-5p. **F** RNA pull-down assay revealed the enrichment of four potential lncRNAs by biotin-labeled miR-330-5p. **G** qRT-PCR determined MIRLET7BHG expression in quiescent and activated HSCs. **H** qRT-PCR validated the depletion efficiency of MIRLET7BHG and also evaluated the influence of silenced MIRLET7BHG on SMO expression in activated HSCs. **I** LncLocator database and subcellular fraction assay revealed the subcellular location of MIRLET7BHG in activated HSCs. **J** Binding sites between MIRLET7BHG and miR-330-5p were predicted by starBase database. **K** RNA pull-down assay revealed the enrichment of MIRLET7BHG pulled down by biotin-labeled miR-330-5p. **L** RIP assay analyzed the enrichment of MIRLET7BHG, miR-330-5p, and SMO in anti-Ago2 group relative to anti-IgG group. **M** Luciferase reporter assay detected the changes in luciferase activity of wild-type and mutant MIRLET7BHG under miR-330-5p overexpression. **P < 0.01, “n.s” represents no significance.

Then, the upstream mechanism of SMO was explored in activated HSCs. We first searched for the miRNAs targeting SMO by starBase (http://starbase.sysu.edu.cn/index.php) database.^30^ Screened by high stringency in CLIP data, miR-330-5p and miR-326 were identified to target SMO. Then, RNA pull-down assay revealed that only miR-330-5p was significantly harvested in the complex pulled down by biotin-labeled SMO (Fig. 4A). Besides, we found that miR-330-5p level was evidently boosted in LX2 cells when activated by exosomes from HCC cells (Figure S2F). Then, we enhanced the expression of miR-330-5p (Fig. 4B) and identified that upregulating miR-330-5p significantly reduced the expression of SMO in exosomes-activated LX2 cells (Fig. 4C). The potential binding sites between SMO and miR-330-5p were obtained from starBase database (Fig. 4D). We mutated the binding sites to explore whether the sites were responsible for the interaction between SMO and miR-330-5p. As revealed by luciferase reporter assay, the luciferase activity of wild-type SMO was significantly reduced by overexpression of miR-330-5p while mutation abrogated such reduction (Fig. 4E).

Next, the potential lncRNAs bound to miR-330-5p were further searched by starBase database. Following the conditions of strict stringency in CLIP data and at least eight cancer types in Pan-Cancer, LRRC75A-AS1, SNHG1, NEAT1, and MIRLET7BHG were predicted to bind with miR-330-5p. According to the results of RNA pull-down assay, only MIRLET7BHG, but not other three candidates, was significantly concentrated in the compounds pulled down by biotin-labeled miR-330-5p (Fig. 4F). Besides, MIRLET7BHG expression was upregulated in activated HSCs than in quiescent HSCs (Fig. 4G), whereas the expression of other three lncRNAs exhibited no difference between activated HSCs and quiescent HSCs (Figure S2G). Noticeably, the downregulation of MIRLET7BHG led to a significant decrease in SMO expression in exosomes-activated LX2 cells (Fig. 4H).

Then, we wondered the subcellular distribution of MIRLET7BHG. Based on the prediction of LncLocator database (http://www.csbio.sjtu.edu.cn/bioinf/lncLocator/),^31^ MIRLET7BHG mainly distributed in cytoplasm. We further conducted subcellular fraction assay and verified MIRLET7BHG was mainly a cytoplasmic lncRNA in activated HSCs (Fig. 4I). Subsequently, the binding sites between MIRLET7BHG and miR-330-5p were obtained based on starBase database (Fig. 4J). Not surprisingly, MIRLET7BHG was notably pulled down by biotinylated wild-type miR-330-5p but not by the mutant group (Fig. 4K). In the meantime, RNA immunoprecipitation (RIP) assay revealed that MIRLET7BHG, miR-330-5p, and SMO were significantly enriched in anti-Ago2 group relative to anti-IgG group (Fig. 4L). Furthermore, the outcomes of luciferase reporter assay exhibited that upregulation of miR-330-5p significantly reduced the luciferase activity of wild-type MIRLET7BHG, whereas had no influence on that of mutant MIRLET7BHG (Fig. 4M). Taken together, MIRLET7BHG served as the ceRNA of SMO via endogenously sponging miR-330-5p in activated HSCs.

### MIRLET7BHG activated HSCs via upregulating SMO

**Fig. 5:**
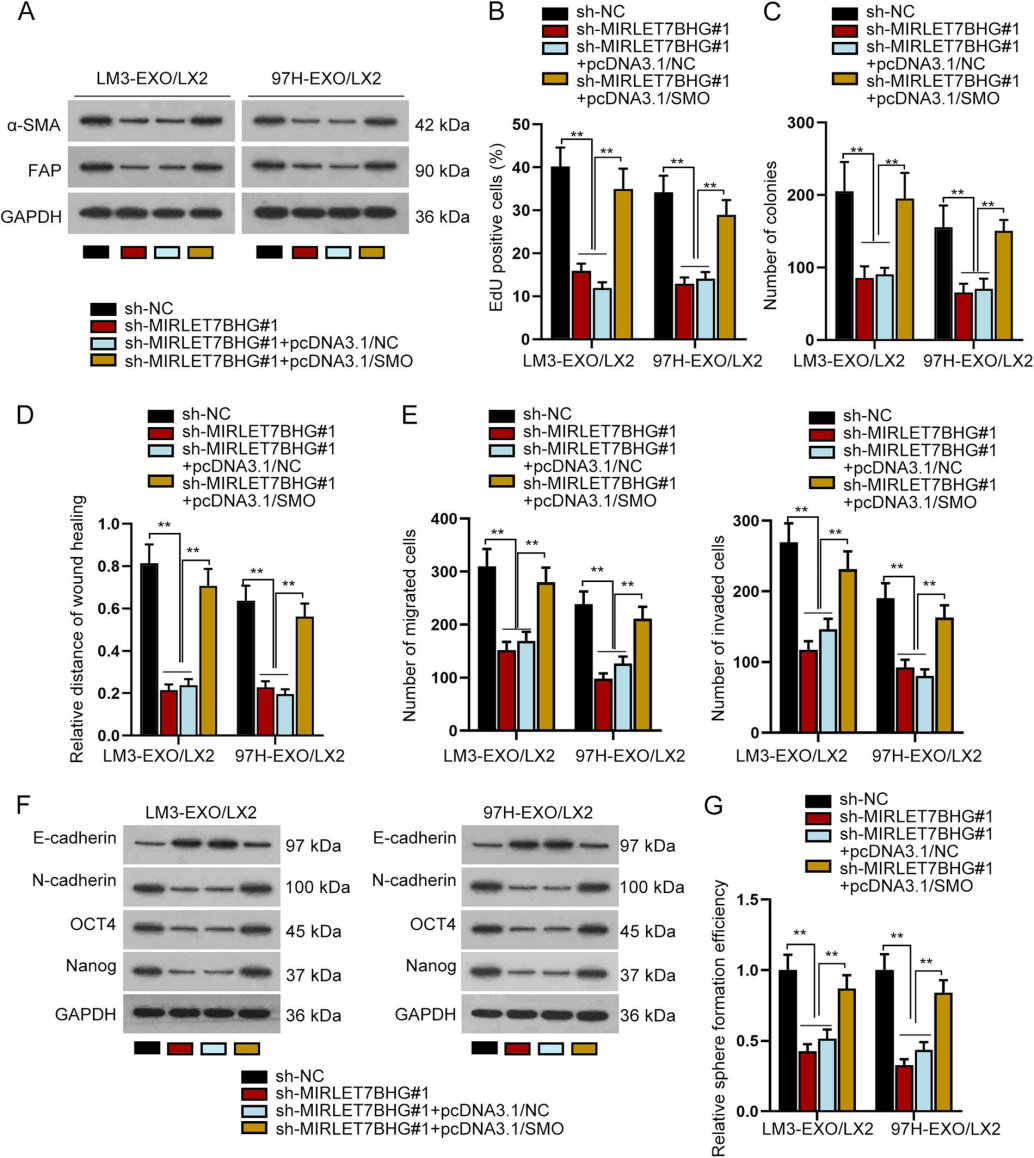
MIRLET7BHG activated HSCs via upregulating SMO. There were four groups of activated HSCs under different transfections: sh-NC, sh-MIRLET7BHG#1, sh-MIRLET7BHG#1+pcDNA3.1/NC, and sh-MIRLET7BHG#1+pcDNA3.1/SMO. **A** Western blot detected the protein level of α-SMA and FAP in different groups. **B**–**C** EdU and colony formation assays examined the proliferation of activated HSCs under different contexts. **D**–**E** Wound-healing and transwell assays analyzed cell migration and invasion capacity in different groups. **F**–**G** Western blot and sphere formation assays analyzed changes in EMT process and stemness of activated HSCs in face of indicated transfections. **P < 0.01.

After verifying the ceRNA mechanism of MIRLET7BHG/miR-330-5p/SMO axis, we then planned to certify whether this axis affected the function of activated HSCs. As revealed by western blot data, MIRLET7BHG knockdown obviously lowered α-SMA and FAP expression in exosomes-activated LX2 cells, whereas such effects were rescued by upregulation of SMO (Fig. 5A). The results suggested that MIRLET7BHG relied on SMO to activate HSCs. Next, colony formation assay and EdU assay revealed that silencing MIRLET7BHG hampered the proliferation of activated LX2 cells and such effects were completely offset under SMO upregulation (Fig. 5B, C). Wound-healing assay and transwell assay uncovered that upregulation of SMO completely counteracted the suppressive impacts of MIRLET7BHG deficiency on the migration and invasion of activated LX2 cells (Fig. 5D, E). Further, the data of western blot analysis and sphere formation assay disclosed that the inhibition of silenced MIRLET7BHG on EMT process and stemness characteristic in activated LX2 cells were completely counteracted by upregulated SMO (Fig. 5F, G). In sum, MIRLET7BHG contributed to HSC activation and strengthened the oncogenic phenotypes of activated HSC via upregulating SMO.

### Gli1 transcriptionally activated MIRLET7BHG

**Fig. 6:**
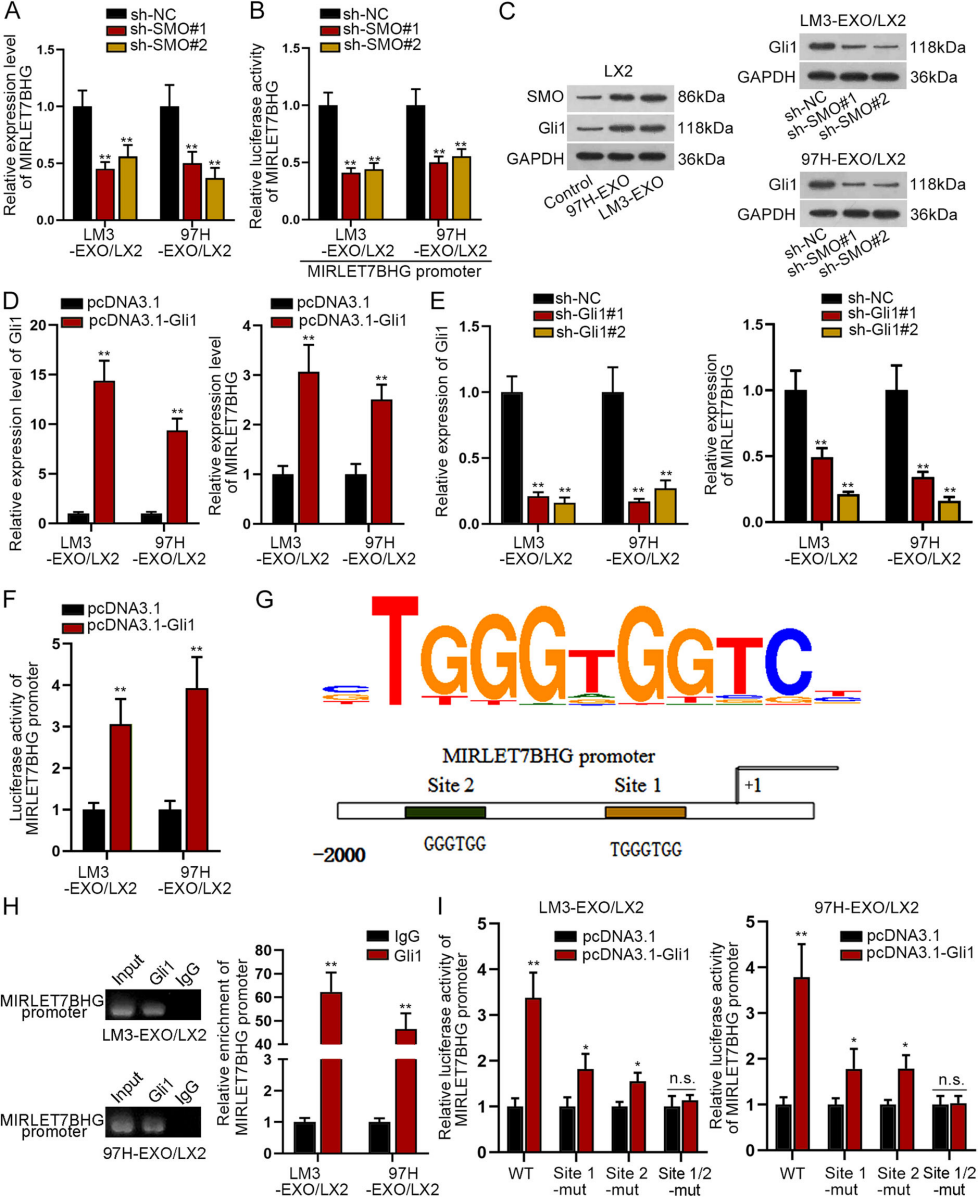
Gli1 transcriptionally activated MIRLET7BHG. **A** qRT-PCR revealed the influence of silenced SMO on MIRLET7BHG expression. **B** Luciferase reporter assay disclosed the luciferase activity of MIRLET7BHG promoter when SMO was subjected to downregulation. **C** Western blot tested hedgehog pathway-associated proteins (SMO and Gli1) in indicated groups. **D** qRT-PCR verified the overexpression efficiency of Gli1 and the influence of upregulated Gli1 on MIRLET7BHG expression. **E** qRT-PCR tested the knockdown efficiency of Gli1 and the influence of downregulated Gli1 on MIRLET7BHG expression. **F** Luciferase reporter assay analyzed the luciferase activity of MIRLET7BHG promoter in activated HSCs with or without the upregulation of Gli1. **G** DNA motif of Gli1 and two binding sites of Gli1 on MIRLET7BHG promoter. **H** ChIP assay revealed the enrichment of MIRLET7BHG promoter in anti-Gli1 group relative to control anti-IgG group. **I** Luciferase reporter assay determined the impact of Gli1 overexpression on the luciferase activity of reporters with indicated MIRLET7BHG promoter (wild-type, site 1-mutated type, site two-mutated type, or site 1/2-mutated type). *P < 0.05, **P < 0.01, “n.s” represents no significance.

Moreover, we detected the impact of SMO on MIRLET7BHG. It was disclosed that loss of SMO significantly reduced the expression of MIRLET7BHG in exosomes-activated LX2 cells (Fig. 6A). According to Fig. 6B, knockdown of SMO caused the suppressed luciferase activity of MIRLET7BHG promoter. On this basis, we speculated that SMO might regulate MIRLET7BH transcription. As we know, SMO has no direct influence on gene transcription. However, it is a crucial protein involved in Hedgehog pathway.^26^ Besides, the expression of Gli1 could be strengthened by upregulation of SMO.^32^ Hence, we wondered if SMO activated the downstream genes of Hedgehog pathway, which further affected the transcription of MIRLET7BHG. So, we then examined the expression of Hedgehog pathway-associated proteins. It was revealed that when LX2 cells were activated by exosomes, the protein level of SMO was significantly enhanced, resulting in elevated expression of Gli1 at both mRNA and protein levels (Fig. 6C and S2H), indicating the activation of Hedgehog pathway under such contexts. In addition, when SMO was knocked down in the activated LX2 cells, Gli1 expression was then decreased (Fig. 6C). Based on these data, we guessed that Gli1 might be the transcription factor of MIRLET7BHG in activated HSCs. To verify this, we enhanced Gli1 expression and found out that upregulation of Gli1 augmented the expression of MIRLET7BHG in activated LX2 cells (Fig. 6D). In reverse, depletion of Gli1 reduced the expression of MIRLET7BHG in such cells (Fig. 6E). Further, luciferase activity of MIRLET7BHG promoter was significantly enhanced in response to the upregulation of Gli1 (Fig. 6F). The DNA-binding motif of Gli1 was obtained from footprintDB database (http://floresta.eead.csic.es/footprintdb/), and two binding sites of Gli1 on MIRLET7BHG promoter were predicted (Fig. 6G). The following ChIP assay revealed that MIRLET7BHG promoter was significantly enriched in anti-Gli1 group (Fig. 6H), proofing the interactivity of Gli1 with MIRLET7BHG promoter in activated LX2 cells. Next, we unveiled that the luciferase activity of MIRLET7BHG promoter was remarkably elevated owing to Gli1 overexpression. Nevertheless, when site 1 or site 2 was mutated, the elevation in the luciferase activity of MIRLET7BHG promoter was slightly mitigated; When both site 1 and site 2 were mutated, the luciferase activity of MIRLET7BHG promoter could not be affected by upregulated Gli1 (Fig. 6I). These results indicated that Gli1 bound to MIRLET7BHG promoter at both sites. On the whole, SMO enhanced Gli1 facilitated the transcription of MIRLET7BHG in activated HSCs.

### MIRLET7BHG mediated HSC activation via Hedgehog pathway

**Fig. 7:**
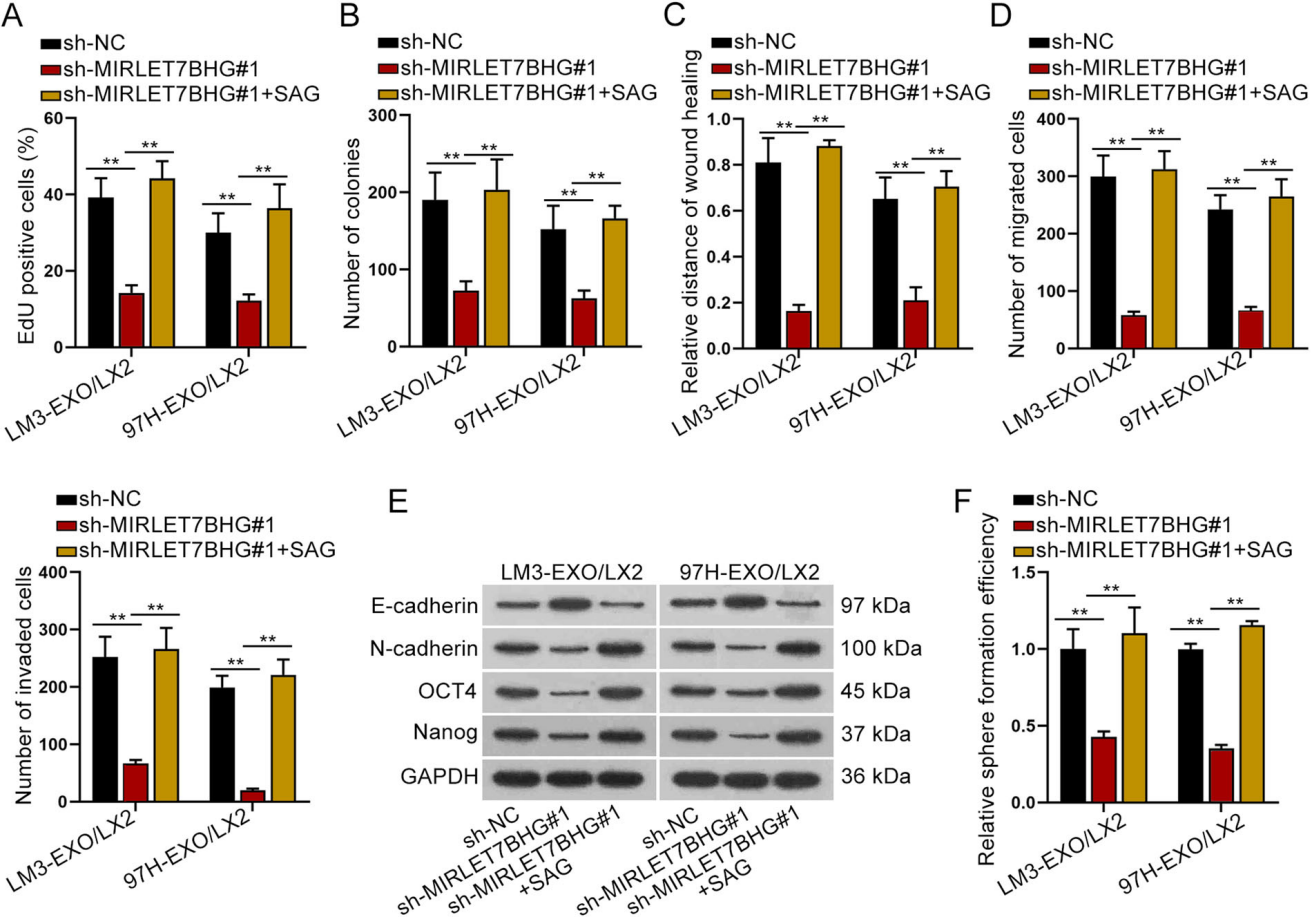
SMO mediated HSC activation via Hedgehog pathway. There were three groups of activated HSCs with different treatments: sh-NC, sh-MIRLET7BHG#1, and sh-MIRLET7BHG#1+ SAG. **A**–**B** EDU and colony formation assays assessed the rescue effects of SAG on MIRLET7BHG deficiency-affected cell proliferation. **C**–**D** Wound-healing and transwell assays monitored cell migration and invasion in different groups. **E**–**F** Western blot and sphere formation assay evaluated the influences of SAG on MIRLET7BHG depletion-hampered EMT and stemness in activated HSCs. **P < 0.01.

In this section, we explored whether Hedgehog pathway was required in MIRLET7BHG-mediated functions in activated HSCs. The activator of Hedgehog pathway, SAG,^33^ was used in the following rescue assays. As demonstrated in Fig. 7A, B, SAG treatment completely rescued the restraining impacts of silenced MIRLET7BHG on cell proliferation. Wound-healing and transwell assays depicted that the suppressive effects of silenced MIRLET7BHG on cell migration and invasion were completely offset by SAG (Fig. 7C, D). Furthermore, SAG completely counteracted the repressive influences of downregulated MIRLET7BHG on EMT process and stemness characteristic (Fig. 7E, F). In conclusion, MIRLET7BHG activated SMO-involved Hedgehog pathway to promote HSC cell proliferation, migration, invasion, EMT, and stemness.

### SMO promoted tumor growth in vivo by activating HSCs

**Fig. 8:**
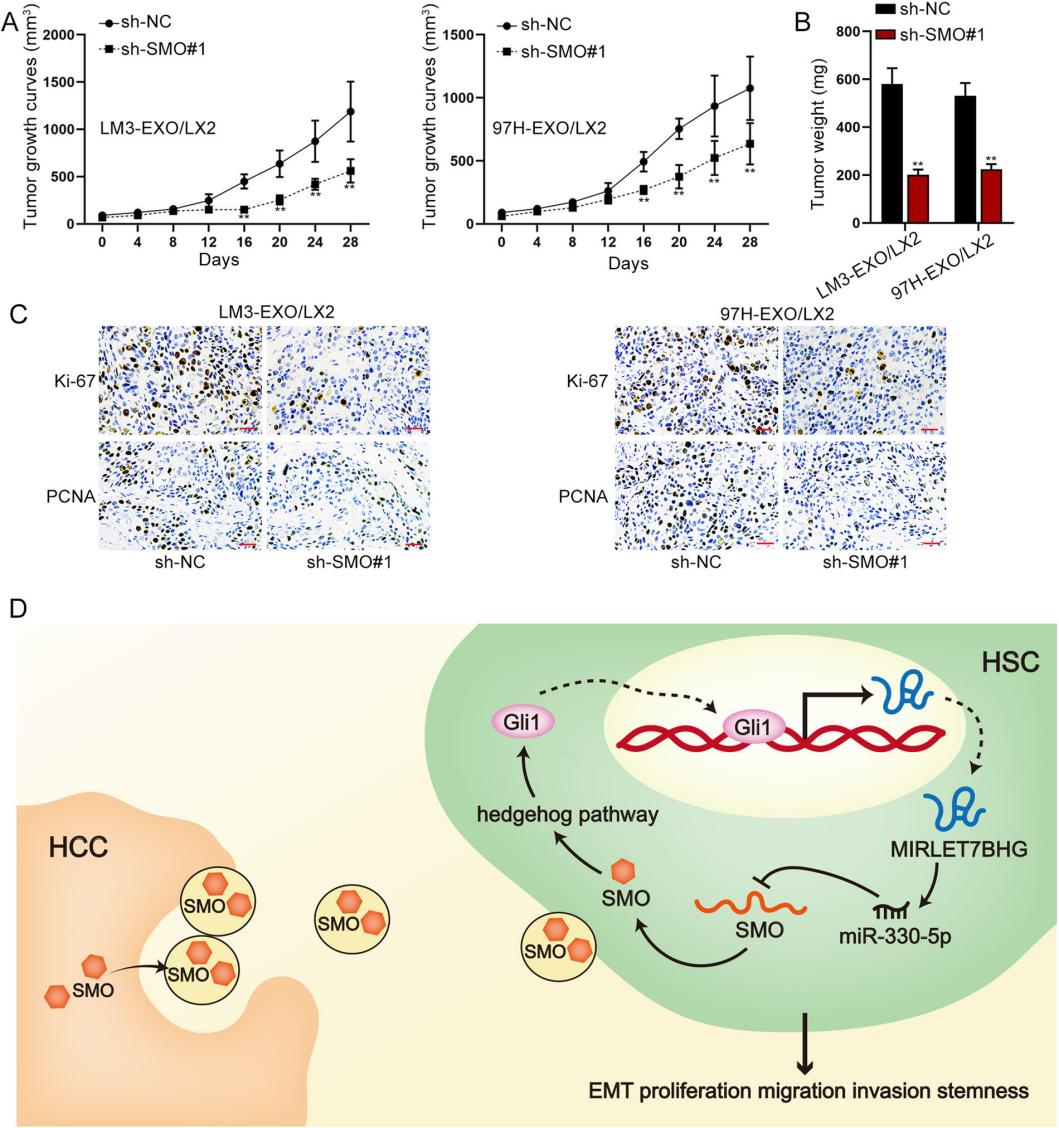
SMO promoted tumor growth in vivo via activating HSCs. **A**–**B** Tumor volume and weight in different groups. **C** IHC staining determined Ki-67 and PCNA positivity in tumors from different groups (bar value = 50 μm). **D** Concept map: Gli1-induced MIRLET7BHG promotes HCC development by activating HSCs through exosomal SMO to trigger the Hedgehog pathway. **P < 0.01.

At last, the in vivo assays were carried out. The xenograft tumor volume and weight were assessed. Results demonstrated that with the depletion of SMO, tumor growth rate was slowed down and the final tumor weight was then reduced compared with control groups (Fig. 8A, B). Data from IHC assay verified similar results that SMO deficiency hindered tumor growth, as the positivity of two proliferative markers (Ki-67 and PCNA) was markedly reduced in tumors with silenced SMO (Fig. 8C). In conclusion, HCC cells-derived exosomes transmitted SMO to HSCs, and SMO activated the Hedgehog pathway to promote HSC cell proliferation, migration, invasion, EMT, and stemness, resulting in HSC activation. Then, in activated HSCs, MIRLET7BHG sponged miR-330-5p to upregulate SMO, further accelerating HCC development. In return, Gli1, the downstream of SMO in Hedgehog pathway, transcriptionally activated MIRLET7BHG in activated HSCs (Fig. 8D).

## Discussion

Activation of HSCs has a close association with HCC occurrence and development.^34,35^ This study first uncovered that SMO was upregulated in HCC cells than in HSCs. Depletion of SMO inhibited HCC cell proliferation, migration, invasion, and EMT. Then, we used HCC cells as donor cells to co-culture HSCs and identified that SMO expression in HSCs was upregulated after co-culture. Further, it was revealed that the co-cultured HSCs exhibited enhanced proliferation, migration, invasion, and EMT than control HSCs, indicating the transfer of HSCs from quiescent stage to activated stage. As exosomes are important for cell-to-cell communication, we supposed if exosomes derived from HCC cells led to the activation of HSCs. As anticipated, we identified that exosomes transmitted SMO from HCC cells to HSCs. The exosomal SMO activated HSCs via promoting HSC proliferation, migration, invasion, and EMT. Previously, Liu R et al. depicted that cholangiocyte-derived exosomal lncRNA H19 facilitates HSC activation and aggravates cholestatic liver fibrosis.^36^ Kim JH et al.^37^ disclosed that miR-192 is transmitted via exosomes from HCV-replicating hepatocytes into HSCs, resulting in the activation of HSCs.

Next, the upstream mechanism of SMO was explored. MIRLET7BHG was discovered to serve as the ceRNA of SMO by competitively binding with miR-330-5p. MIRLET7BHG is seldom studied in human cancers. The only report is that MIRLET7BHG polymorphisms are potential predictive markers for asbestos exposure-associated lung cancer.^38^ MiR-330-5p was widely reported as a tumor suppressor.^39^ Also, ceRNA network involving miR-330-5p has already been reported in a former study. LINC00958 binds with miR-330-5p to upregulate PAX8 in a competitive fashion, eventually contributing to pancreatic cancer progression.^40^ This study first uncovered that MIRLET7BHG contributed to the activation of HSCs by upregulating SMO via a miR-330-5p-dependent way. Future work will be required to examine the function of miR-330-5p in HSC activation and HCC development. Apart from the present study, the ceRNA networks implicated in HSCs activation have been commonly proposed. Yu F et al.^23^ revealed that lncRNA SNHG7 promotes the activation of HSCs via endogenously sponging miR-378a-3p to elevate DVL2 expression. Li Z et al. uncovered that HOTTIP facilitates the progression of liver fibrosis by activating HSCs by upregulating TGFBR1 and TGFBR2 in a miR-148a-dependent manner.^41^

Hedgehog pathway is a common oncogenic pathway and could promote the myofibroblastic phenotype in HSCs.^42^ In the current study, we verified that SMO activated Hedgehog pathway. Hedgehog signaling is generally triggered via the binding of the Hedgehog ligand to PTCH1, which is a negative regulator of SMO.^43^ After the binding of Hedgehog ligand to PTCH1, SMO is released and then stimulates a cascade to promote the nuclear translocation of the downstream Gli transcription factors. Subsequently, the activated Gli transcription factors induce the expression of diverse target genes.^44^ Regardless of ligand-dependent or ligand-independent mechanism, aberrant activation of Hedgehog pathway always triggers Gli transcription factors. Thus, Glis play a central role in this pathway. This study first uncovered that Gli1 transcriptionally activated MIRLET7BHG in HSCs, thus promoting HSC activation and HCC tumor growth. Besides, the role of Gli1 in HCC has been largely unveiled. For instance, lncRNA LINC01093 impairs HCC progression by binding with IGF2BP1 to facilitate the degradation of Gli1 mRNA (messenger RNA).^45^ PCAF antagonizes the effects of Gli1 on inducing EMT and tumor metastasis in HCC.^46^

In conclusion, our current study demonstrated that SMO was transmitted from HCC cells to HSCs via exosomes. MIRLET7BHG activated HSCs by enhancing HSC proliferation, migration, invasion, and EMT by sponging miR-330-5p to upregulate SMO. In turn, SMO induced Hedgehog pathway and indirectly facilitated the activating effects of Gli1 on MIRLET7BHG transcription. Finally, activated HSCs contributed to HCC development. The findings above might shed new insight into HCC treatment.

## Supplementary information

Supplementary Figure legends

Figure S1

Figure S2
