# Supplementary material for: MIRLET7BHG promotes hepatocellular carcinoma progression by activating hepatic stellate cells through exosomal SMO to trigger Hedgehog pathway
Source: Cell Death Dis. 2021 Mar 26;12(4):326. doi: 10.1038/s41419-021-03494-1 (PMC7997896; doi:10.1038/s41419-021-03494-1)
Supplement: Supplementary file 1 — Supplementary Figure legends [file 41419_2021_3494_MOESM1_ESM.docx]

**Figure S1.**

**A-B**. Colony formation and EDU assays examined LX2 cell proliferation with or without co-culture with conditioned medium (CM) of LM3 or 97H cells. **C-D**. Wound healing and transwell assays assessed the migratory and invasive capacity of LX2 cells with or without co-culture. **E-F**. Western blot and sphere formation assay evaluated EMT process and stemness characteristic in LX2 cells under the treatment of CM from LM3 or 97H cells. ^**^P<0.01.

**Figure S2.**

**A**. Flow cytometry analyzed the size of exosomes from HCC cells. **B**. The expression of SMO in LX2 cells treated with increasing doses of HCC cells-derived exosomes was analyzed via qRT-PCR. **C**. The levels of COL1A1 and COL4A1 in control LX2 cells (quiescent HSCs) and exosomes-treated LX2 cells (activated HSCs) were detected by qRT-PCR. **D**. ELISA assay analyzed the secretion of pro-collagen type I α1 in quiescent and activated HSCs. **E**. Western blot tested the level of collagen I in quiescent and activated HSCs. **F**. qRT-PCR determined miR-330-5p expression in activated HSCs relative to quiescent controls. **G**. The expression of LRRC75A-AS1, SNHG1 and NEAT1 in quiescent and activated HSCs was detected through qRT-PCR. **H**. qRT-PCR determined Gli1 expression in activated HSCs relative to quiescent controls. ^*^P<0.05, ^**^P<0.01, “n.s” represents no significance.
