# Supplementary figures and images for: MIRLET7BHG promotes hepatocellular carcinoma progression by activating hepatic stellate cells through exosomal SMO to trigger Hedgehog pathway
Source: Cell Death Dis. 2021 Mar 26;12(4):326. doi: 10.1038/s41419-021-03494-1 (PMC7997896; doi:10.1038/s41419-021-03494-1)

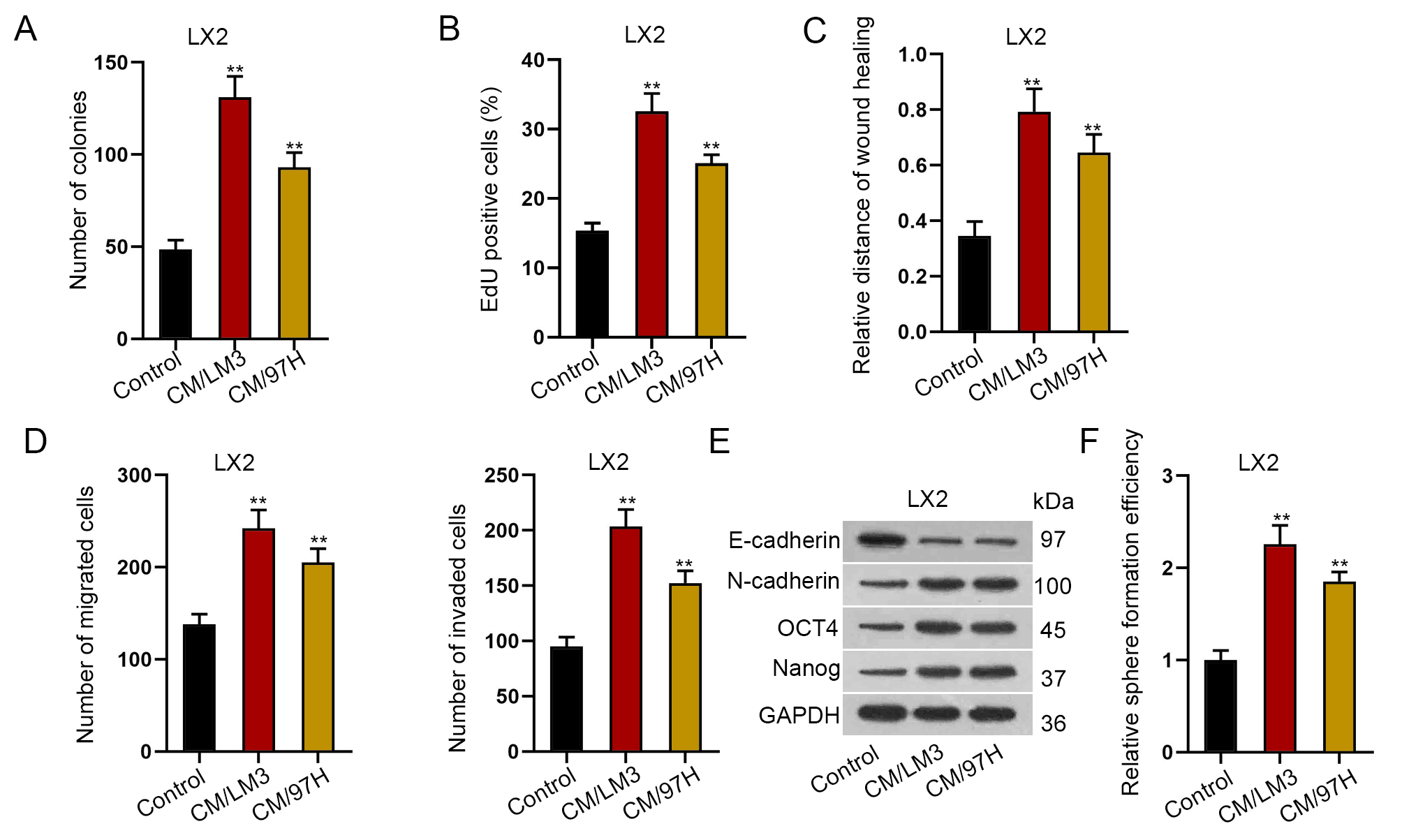

Supplement: Supplementary file 2 — Figure S1 [file 41419_2021_3494_MOESM2_ESM.tif]

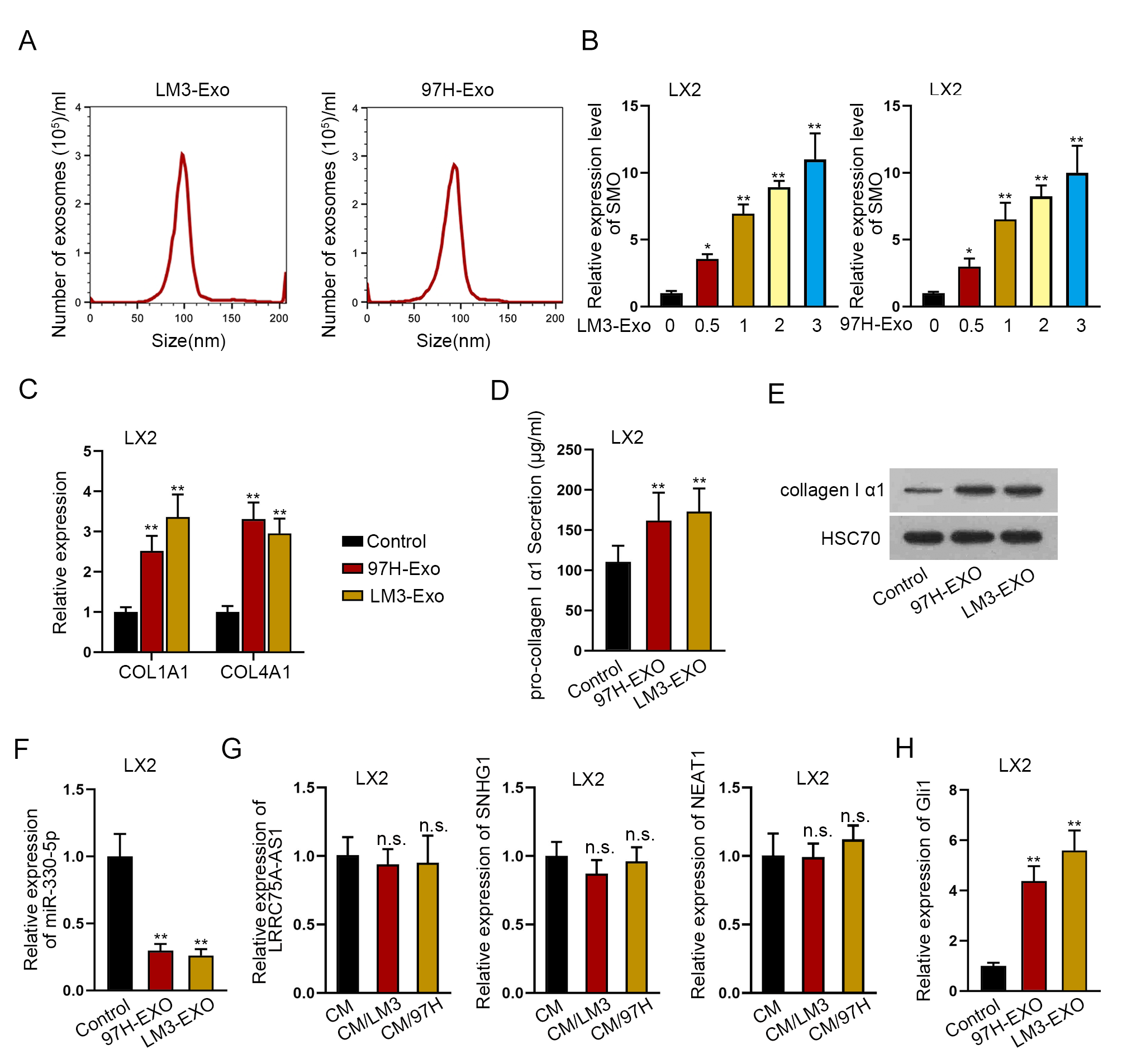

Supplement: Supplementary file 3 — Figure S2 [file 41419_2021_3494_MOESM3_ESM.tif]
